# Oseltamivir Resistance in Influenza A(H6N2) Caused by an R292K Substitution in Neuraminidase Is Not Maintained in Mallards without Drug Pressure

**DOI:** 10.1371/journal.pone.0139415

**Published:** 2015-09-30

**Authors:** Anna Gillman, Shaman Muradrasoli, Andreas Mårdnäs, Hanna Söderström, Ganna Fedorova, Max Löwenthal, Michelle Wille, Annika Daggfeldt, Josef D. Järhult

**Affiliations:** 1 Section for Infectious Diseases, Department of Medical Sciences, Uppsala University, Uppsala, Sweden; 2 Zoonosis Science Centre, Department of Medical Biochemistry and Microbiology, Uppsala University, Uppsala, Sweden; 3 Department of Biomedical Sciences and Veterinary Public Health, Swedish University of Agricultural Sciences, Uppsala, Sweden; 4 Department of Chemistry, Umeå University, Umeå, Sweden; 5 Faculty of Fisheries and Protection of Waters, South Bohemian Research Center of Aquaculture and Biodiversity of Hydrocenoses, Vodnany, University of South Bohemia in Ceske Budejovice, Ceske Budejovice, Czech Republic; 6 Centre for Ecology and Evolution in Microbial Model Systems, Linnaeus University, Kalmar, Sweden; 7 Department of Virology, Immunobiology and Parasitology, Swedish Veterinary Institute, Uppsala, Sweden; Boston University School of Medicine, UNITED STATES

## Abstract

**Background:**

Wild waterfowl is the natural reservoir of influenza A virus (IAV); hosted viruses are very variable and provide a source for genetic segments which can reassort with poultry or mammalian adapted IAVs to generate novel species crossing viruses. Additionally, wild waterfowl act as a reservoir for highly pathogenic IAVs. Exposure of wild birds to the antiviral drug oseltamivir may occur in the environment as its active metabolite can be released from sewage treatment plants to river water. Resistance to oseltamivir, or to other neuraminidase inhibitors (NAIs), in IAVs of wild waterfowl has not been extensively studied.

**Aim and Methods:**

In a previous *in vivo* Mallard experiment, an influenza A(H6N2) virus developed oseltamivir resistance by the R292K substitution in the neuraminidase (NA), when the birds were exposed to oseltamivir. In this study we tested if the resistance could be maintained in Mallards without drug exposure. Three variants of resistant H6N2/R292K virus were each propagated during 17 days in five successive pairs of naïve Mallards, while oseltamivir exposure was decreased and removed. Daily fecal samples were analyzed for viral presence, genotype and phenotype.

**Results and Conclusion:**

Within three days without drug exposure no resistant viruses could be detected by NA sequencing, which was confirmed by functional NAI sensitivity testing. We conclude that this resistant N2 virus could not compete in fitness with wild type subpopulations without oseltamivir drug pressure, and thus has no potential to circulate among wild birds. The results of this study contrast to previous observations of drug induced resistance in an avian H1N1 virus, which was maintained also without drug exposure in Mallards. Experimental observations on persistence of NAI resistance in avian IAVs resemble NAI resistance seen in human IAVs, in which resistant N2 subtypes do not circulate, while N1 subtypes with permissive mutations can circulate without drug pressure. We speculate that the phylogenetic group N1 NAs may easier compensate for NAI resistance than group N2 NAs, though further studies are needed to confirm such conclusions.

## Background

Antiviral resistance of human influenza viruses is monitored by surveillance of clinical samples. Currently, over 90% of circulating human influenza A viruses (IAVs) (H3N2, H1N1pdm09) is amantadine resistant worldwide, leaving neuraminidase inhibitors (NAIs), primarily oseltamivir, the drug of choice for treatment of severe influenza infections [1,2]. Recent insights in the complex dynamics of NAI resistance and related compensations for reduced fitness are primarily the result of studies on clinical isolates [3–5]. The NA amino acid substitutions that generate resistance to NAIs are subtype-specific, as the two phylogenetic NA groups N1 (including N1, N4, N5, N8) and N2 (including N2, N3, N6, N7, N9) differ in structure and substrate binding; in N1 viruses the H274Y (N2 numbering) substitution is most common, while in N2 viruses R292K or E119V are most common [5–7].

The natural reservoir hosts of IAVs are wild waterfowl [8,9]. These wild migratory birds can host subtypes with most combinations of 16 hemagglutinin (HA) and 9 neuraminidase (NA) surface proteins [10], and disperse viruses along migratory routes. The migration allows mixing of naïve and infected birds, transmission of multiple viruses simultaneously and generation of hetero- and homosubtypic immunity [8,11]. In addition, separate flyways lead to circulation of multiple homosubtypic strains [8]. The ecology and immunity of wild waterfowl combined with the segmented IAV genome and the low fidelity IAV polymerase complex [12] result in continuous point mutations and reassortment of the genetic segments [10,13]. Consequently, the variability of waterfowl viruses is large and exceeds that observed in IAVs infecting other species [9]. As wild waterfowl provide a reservoir for highly pathogenic IAVs, and is a source for genetic segments which can reassort with poultry or mammalian adapted IAVs to generate novel viruses [14], knowledge on the potential for NAI resistance in IAVs of wild waterfowl is of interest. Exposure of wild birds to NAIs may occur in the environment as active drug metabolites are released from sewage treatment plants, via treated sewage water, to downstream rivers [15–21]. To date, resistance surveillance in wild bird avian IAVs has been limited, a few studies from North America and northern Europe have not detected circulating resistant viruses [22–24]. NAI resistance substitutions in poultry adapted avian IAVs, detected in treated humans, are also subtype-specific, with R292K or E119V most usual in H7N9 viruses [25] and H274Y in H5N1 viruses [26,27]. In an H10N8 virus though, despite classed as group N1 neuraminidase, NAI treatment selected for the R292K substitution [28].

In order to investigate the propensity of wild bird avian IAVs to acquire NAI resistance if the natural host birds are exposed to active drug metabolites in their water, an *in vivo* Mallard *(Anas platyrhynchos)* model, in which the dynamics of NAI resistance can be explored, was previously developed. The H274Y NA substitution was induced in a Mallard H1N1 virus by oseltamivir exposure, and was retained when drug exposure was removed, i.e. the resistant virus may have the potential to circulate among wild birds [29,30]. When Mallards were infected with H6N2 virus and exposed to oseltamivir the virus became resistant by the R292K substitution [31]. The resistant H6N2/R292K virus infected and transmitted equal to wild type virus in Mallards and the resistance was retained through drug free chicken egg propagation, indicating a retained viral fitness. In addition to the R292K substitution, also the D113N and D141N substitutions of unknown significance were observed in NA [31]. In the present study we tested if the resistant H6N2/R292K virus could maintain the resistance in Mallards without drug exposure, i.e. if viral fitness could be retained despite the NA substitution. In addition we aimed to determine a possible fitness modulating effect by the D113N or D141N substitutions.

## Methods

### Ethics Statement

This study was carried out in strict accordance with the recommendations for care and use of laboratory animals of the Swedish Board of Agriculture. The protocol was approved by the Ethics Committee on Animal Experiments in Uppsala, Sweden (permit C63/13). Birds were euthanized with sodium pentobarbital (by intravenous injection of 100 mg/kg [Pentobarbital vet. 100 mg/mL]).

### Virus

Three different isolates of oseltamivir resistant influenza A(H6N2) virus containing the NA amino acid substitution R292K (N2 numbering) were used. The variants had evolved during an experimental *in vivo* study by exposure of infected Mallards with the active metabolite of oseltamivir, oseltamivir carboxylate (OC) [31]; the used A/Mallard/Sweden/50908/2006(H6N2) virus (NA accession number AFV33710) had previously been isolated and typed during IAV surveillance studies [32]. The isolates for this study were obtained from experimental fecal samples by isolation in 11 days old specific pathogen free (SPF) embryonated chicken eggs (ECE). Samples were inoculated in the ECE allantoic cavity and harvested after two days; the presence of IAV was confirmed with turkey erythrocytes hemagglutination. If negative, a second SPF ECE passage was done. The allantoic harvests were used as viral stock solution for inoculation in the experiments. The NA sequences of the isolates were verified by sequencing of the NA gene (see below under viral detection and sequencing of NA), i.e. viruses used in the experiments had the NA amino acid variants R292K, or R292K plus D113N, or R292K plus D141N.

### Drugs

OC and deuterium-labeled OC were obtained from F. Hoffmann—La Roche Ltd (Basel, Switzerland), and Zanamivir (ZA) (for use in the neuraminidase inhibition assay, as described in [33]) was purchased locally as Relenza®, and dissolved in double distilled water. Stock solutions of both compounds were stored at minus 20°C.

### Mallard model

We used a previously described *in vivo* Mallard model [30], which was designed to allow continuous propagation of virus in successively introduced naïve pairs of birds that could transmit the latest evolved viral variants. One day old male Mallards were purchased and bred in isolation indoors at the Swedish Veterinary Institute’s facility and were used in the experiments at three months of age. Housing, animal welfare, and all experiments were done in accordance with legislation and recommendations by the Swedish Board of Agriculture. During breeding and housing 120 birds were kept together in a large room with cycling daylight, swimming pools in which the water was changed daily, dry areas with sawdust or hay and feed *ad libitum*. All birds were cared for daily and observed for gain of weight, clean and dry feathers and natural behavior. In the case (very few) good status was not maintained, birds were euthanized (as described below). Experiments were performed under the same conditions and experimental procedures were set up to minimize suffering, all in close cooperation with the laboratory animal veterinary of the Swedish Veterinary Institute. Before including Mallards in the experiments, IAV infection was ruled out by blood serology (FlockCheck, Avian Influenza Virus Antibody Test Kit, IDEXX, Hoofddorp, The Netherlands) taken at 10 weeks of age, and by real time reverse (RRT) PCR of the IAV matrix gene from fecal samples (described below), taken the day of inclusion in the experiments. Three identical experiments with the three resistant isolates were performed, using five generations of ducks, two individuals in each, over 17 days. Generation one was inoculated at day zero of the experiment in esophagus with 1 mL viral stock solution and placed in an experimental room containing a 170 L water pool, spiked with OC to a concentration of 10 μg/L. The water was changed daily and the drug concentration was reduced during the experiment to 3 μg/L of OC from day two, to 1 μg/L from day five, and from day nine there was no drug exposure. On day three (after approximately 72 hours), generation two was placed in the experimental room together with generation one until day five (during approximately 48 hours) to allow viral transmission, where after generation one was euthanized with sodium pentobarbital (by intravenous injection of 100 mg/kg [Pentobarbital vet. 100 mg/mL]). On day six (approximately 24 hour later), generation three was introduced to the room etc. Fecal samples were collected daily from each bird by placing them in clean boxes for five to 20 minutes, where after swabs were taken from left droppings. Occasionally cloacal swabbing replaced fecal swabs if no feces were left in the box. Swabs were immediately frozen in influenza medium at -80°C until further processing.

### Viral detection and sequencing of NA

Viral RNA was extracted from all fecal samples and from water with a Magnatrix 8000 extraction robot (Magnetic Biosolutions, Stockholm, Sweden) and the Vet viral RNA kit (NorDiag ASA, Oslo, Norway). To improve NA sequence quality some samples were re-extracted from fecal samples with TRIzol® (Life technologies™) using 900 μL reagent per 100 μL sample. Detection and quantification of IAV from extracted RNA was done by RRT-PCR with primers and probe targeting the influenza A matrix gene [34], and iScript one-step RT-PCR kit for probes (Bio-Rad). Reaction volumes of 25 μL with 0.5 μL enzyme mix and final concentrations of primers and probe of 400 nM and 120 nM respectively, were run in a Corbett Research Rotor-Gene 2000 Real-time Thermo Cycler (Corbett Research). Samples with a cycle threshold (CT) value ≥45 were considered negative.

The NA gene was amplified from RNA of fecal samples, in which IAV had been detected by RRT-PCR, with a one-step reverse transcriptase PCR (RT-PCR) using one forward and one reverse primer (Table 1), and SuperScript™ III One-Step RT-PCR System with Platinum® Taq High Fidelity polymerase (Life Technologies). Reaction volumes of 25 μL contained 0.25 μL enzyme mix, final primer concentrations of 400 nM each and 5 μL (1 pg to 1 μg) RNA sample. Thermocycling conditions were 30 min at 55°C and 5 min at 94°C for reverse transcription, followed by 35 amplification cycles of 1 min at 94°C, 1 min at 60°C and 4 min at 68°C. PCR products were confirmed by gel electrophoresis and purified either by enzymatic treatment with ExoSAP-IT (Affymetrix Inc, California, USA), using 2 μL reagent to treat 24 μL sample, or by gel extraction with the QIAquick gel extraction kit (QIAGEN). Purified PCR products were sequenced by Sanger sequencing at Macrogen Inc (The Netherlands) using two forward and two reverse primers (Table 1). Sequence results were analyzed in SeqScape® Software v2.7 (Applied Biosystems®) with the A/Mallard/Sweden/50908/2006(H6N2) NA sequence as a reference.

**Table 1:** Primers for NA amplification and sequencing.

Primer	Sequence (5’-3’)	Location (5’-3’)	Application
H6N2-NA-FW1	TGAACCCAAATCAGAAGATAATAACA	2–27	Amplification, sequencing
H6N2-NA-Rev1	GCGAAAGCTTATATAGGCATGAA	1395–1419	Amplification, sequencing
H6N2-NA-FW2	GTGTGCATAGCATGGTCCAG	520–540	Sequencing
H6N2-NA-Rev2a	AACCTGAGCGTGAATCCTTG	1100–1120	Sequencing

### Phenotypic resistance testing

Five samples with the NA R292K substitution (one or two from each experiment) and six wild type samples (two from each experiment) were tested regarding NA inhibition by OC and zanamivir. To obtain a sufficient viral titer for the assay, fecal samples were propagated in SPF ECE (Valo, Germany), followed by NA sequencing to verify the genotype. Here, RNA was extracted on a Maxwell® 16 instrument with the Maxwell® 16 Viral Total Nucleic Acid Purification Kit (Promega Biotech AB) according to the manufacturer’s instructions. Inhibition of NA by OC and ZA were determined on duplicate samples in a fluorogenic NA substrate assay using 29-(4-methylumbelliferyl)-a-D-N-acetylneuraminic acid (MUNANA; Sigma) and 10 step dilution-series of OC and ZA in black 96-well flat-bottom plates, according to the protocol of the European network for management of drug-resistant viruses Virgil Clinvir [35]. Virus and drugs were pre-incubated at 37°C for 30 minutes followed by 60 minutes incubation with the MUNANA substrate. Fluorescent products were measured in an Infinite® M1000 PRO (Tecan) microplate reader, and drug concentrations that inhibited 50% of viral NA activity (IC_50_) were determined from best fit dose response curves with the Prism6 GraphPad software (GraphPad).

### Quantification of OC in water

One water sample (_~_ 40 mL) from the experimental water pool was collected before change of water each day of the experiments. In addition, for evaluation of the method’s variation and OC degradation during 24 hours, triplicate samples were collected directly after OC was added and after 24 h before the water was changed (0h and 24h samples, respectively), during the first day (exposure level 10 μg/L) of each experiment. Finally, one 24h sample from each experiment was collected from water to which no OC had been added (from day 10 of the experiments). Ten milliliters of each water sample was pre-filtered (0.45 μm) and acidified (0.1% formic acid). The OC concentration in the water was analyzed on one mL of the filtrate using an on-line solid phase extraction (SPE)/liquid chromatography-tandem mass-spectrometry (SPE/LC-MS/MS) method, as previously described [36]. Samples were quantified using the internal standard method (with deuterium labelled OC as internal standard), with a calibration curve in the range of 1–10 μg/L.

### Statistic calculations

Hypothesis testing on difference of IC_50_ values between R292K and wild type samples was done with the non-parametric Mann-Whitney U-test on difference in median values. Hypothesis testing on equal excretion of virus by drug exposed (generation 1 and 2) and unexposed (generation 4 and 5) Mallards by CT values of the RRT-PCR was done with unpaired t-test, and that on equal distribution of genotypes in samples from drug exposed and unexposed Mallards with Fisher’s exact test. All tests were done using the Statistica 12 (StatSoft®) software.

## Results

### Infection and transmission of virus

**Fig 1:**
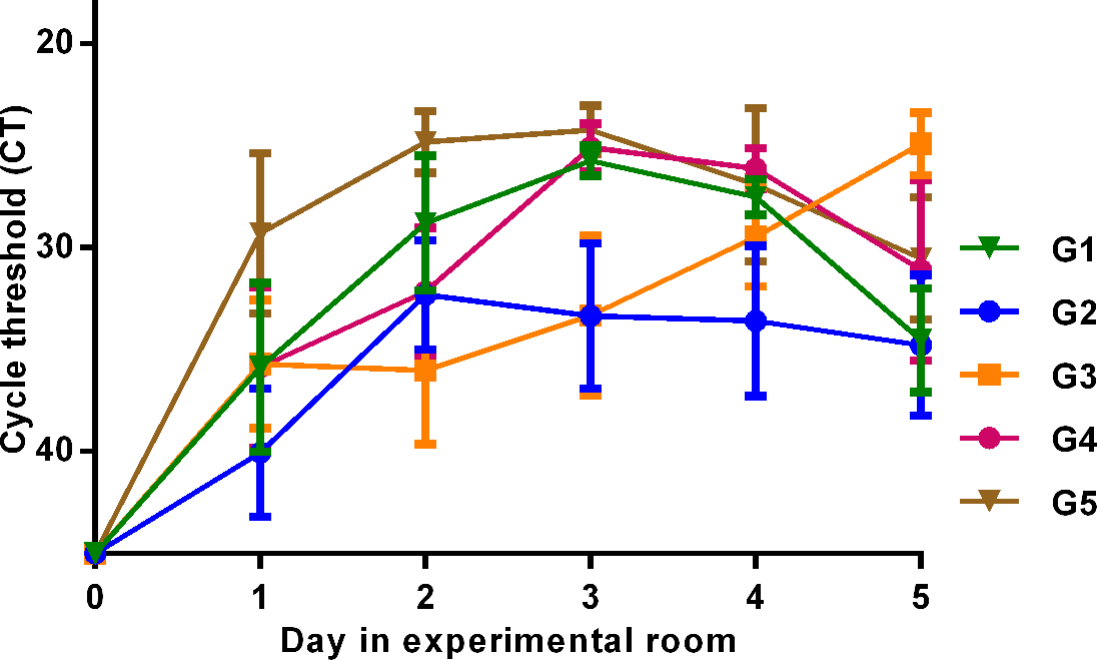
Viral excretion. IAV was detected by RRT-PCR of the matrix gene from daily fecal samples. The Y-axis displays cycle threshold (CT) values as a quantitative measure of viral excretion. Samples with CT values ≥ 45 were considered negative. The X-axis displays which days of the experiment samples were collected from the Mallards. G1 = two birds in each of three experiments (n = 6), etc. G1 and G2 were OC exposed, G3 was OC exposed day 0–2, and G4 and G5 were unexposed. Value points display mean CT values of 6 samples and error bars display standard errors of the mean (SEM). No significant difference in excretion was detected between drug-exposed (G1 and G2 with resistant genotype NA-292K) and unexposed (G4 and G5 with wild type genotype NA-292R) birds day 1, 4 or 5, while drug-exposed birds had lower virus excretion levels day 2 and 3(*P* = 0.034).

The overall results did not differ between the three experiments, with different viral variants. IAV was detected in feces from all Mallards of the experiments. The excretion patterns were over all similar between birds that were inoculated in esophagus and those infected by transmission, as well as between birds that were exposed or unexposed to OC (Fig 1).

### NA sequences of fecal samples

Neuraminidase RNA from thirty-four fecal samples were successfully sequenced, with a minimum of two samples from drug exposed and two from the last generation of unexposed Mallards of each experiment. Lysine (K) was detected at amino acid residue 292 of NA in all viruses isolated from birds exposed to OC. Within one to three days after removal of OC, arginine (R) was detected at NA residue 292 in all birds, where after no 292K could be detected throughout the experiments. The resistant viral variant with only R292K was transmitted to one naïve Mallard after removal of OC, but was not detected the following day (Table 2). The NA substitutions D113N and D141N persisted through the experiments and the ECE propagation process.

**Table 2:** Experimental design and NA sequence results of residue 292.

Day	OC (μg/L)*	G1						G2						G3						G4						G5					
	(%RSD)	E1		E2		E3		E1		E2		E3		E1		E2		E3		E1		E2		E3		E1		E2		E3	
0	10 (4–7%)																														
1																															
2	3 (9–11%)					K																									
3						K	K																								
4																															
5	1 (3–10%)																														
6																															
7									K		K	K																			
8												K			K		K														
9	0																	K													
10															R			R													
11															R		R		K						*k*						
12																						R		R	R						
13																				R	R	R						R			
14																				R		R		R		R				R	R
15																											R	R		R	
16																											R		R		
17																														R	

The experiment was repeated three times with three H6N2/R292K resistant IAV variants, each consisting of five overlapping generations of Mallards, two birds in each.

*Oseltamivir carboxylate (OC) concentration in the experimental water with range of relative standard deviations (%RSD) for each of the three experiments at respective exposure level (n = 4 at 10 μg/L, n = 3 at 3 μg/L and n = 4 at 1 μg/L). The three experiments had the same OC concentration in the water. Dashed horizontal lines indicate change in OC concentrations.

G1 = generation one, G2 = generation two etc.

E1 = experiment with NA substitutions R292K + D113N. E2 = experiment with NA substitutions R292K + D141N. E3 = experiment with NA substitutions R292K. K displays lysine, R displays arginine, and *k* displays a mixed proportion of R and K at NA residue 292, as determined by Sanger sequencing of daily fecal samples of each bird. Wild type genotype correlated to unexposed Mallards (*P* = 0.000002)

### Phenotypic NAI sensitivity testing

Median IC_50_s by OC and ZA differed between wild type and R292K mutated virus (*P* = 0.008 for both). Mean IC_50_ values are displayed in Table 3 and correspond to 15,000 fold and 10 fold increased IC_50_s for the R292K mutant by OC and zanamivir respectively. Presence of D113N or D141N did not influence the results.

**Table 3:** Viral NA inhibition by oseltamivir carboxylate and zanamivir.

Virus	Oseltamivir carboxylate	Zanamivir
	IC_50_ ± SD (nM)	IC_50_ ± SD (nM)
**292R** (n = 6)	0.19 ± 0.12	0.37 ± 0.13
**292K** (n = 5)	2,900 ± 410	3.7 ± 1.5

Mean concentrations of drugs ± standard deviations that inhibited 50% (IC_50_) of the viral NA activity in a functional assay with the MUNANA substrate. 292R = arginine at NA residue 292. 292K = lysine at NA residue 292. Values were calculated on duplicates of six wild type (292R) and five mutated (292K) viral samples.

### OC concentrations in experimental water

The limit of quantification (LOQ) of the OC analysis was 0.0025 μg/L, the linearity (R2) of the calibration curve (0.001–10 μg/L range) was 0.9999, and the relative standard deviation for triplicates for 0h and 24h samples ranged from 0% to 11% for the three experiments. The mean OC concentrations, calculated for each experiment and from all 24h samples, including the triplicates of the first day of exposure, were the same for all three experiment (1, 3 and 10 μg/L) with relative standard deviations (%RSD) in the range 3–11%, and are shown in Table 2.

The difference in mean OC concentrations between 0h samples and 24h samples were within expected variations of the methods, with 4% to 8% increase during 24h. Samples collected 24 hours after removed drug exposure (one sample from each experiment) contained no OC.

## Discussion

In this study, where we aimed to approximate natural infection and transmission of an oseltamivir resistant influenza A(H6N2) virus in the natural wild bird host, we observed that the resistance substitution R292K in NA could not be maintained without drug pressure. Although resistant virus could readily infect and transmit between Mallards to a similar degree as wild type virus, the mutant was outcompeted by wild type virus within one to three days in all birds unexposed to OC. The reversion to wild type virus by NA genotype determination was confirmed with phenotypic resistance testing. The reverted wild type virus had similar IC_50_ values as the original virus collected from a wild Mallard, and which had been used in the previous OC exposure study [31]. The D113N and D141N substitutions, which had emerged in parallel to R292K in the previous OC exposure experiment [31], did not influence the time to reversal to wild type virus, interpreted as having no significant fitness related effects on the resistant virus. It is possible that their occurrence was not related to OC exposure, as the variants are commonly found in avian N2 neuraminidases [31].

The genetic variability of avian IAVs is large [10,13,14] and the propensity to acquire and retain resistance mutations vary between and within subtypes, related both to NA-group specific effects at the enzyme binding site [5,37] and to the presence of compensatory or permissive additional mutations [4,38]. Among human IAVs, oseltamivir resistant H1N1/H274Y viruses with a suitable genetic context can circulate without drug pressure [4,38], but to date no circulation of resistant human H3N2 viruses has been seen [1]. In avian IAVs, it was previously observed *in vivo* that an H1N1/H274Y virus, which had acquired resistance by OC exposure in Mallards, maintained the resistance substitution without drug pressure [30]. This contrasts to the results of the present study, in which the resistant H6N2/R292K virus was rapidly outcompeted by wild type virus when drug pressure was removed from infected Mallards. Thus, both occurrence of resistance in circulating human strains and *in vivo* experimental data from one N1 and one N2 subtype IAV suggest that resistance may be better harbored without fitness loss in an N1 than in an N2 subtype. As N1 and N2 belong to different phylogenetic groups of NA [7] it is tempting to suggest that group N1 NA-containing viruses more easily can compensate for resistance related loss of fitness, and therefore more easily retain resistance without drug pressure, than viruses with group N2 NAs. However, few strains and subtypes have been observed and examined and extended assessment is needed for a definite conclusion.
